# XML Attenuates Ox‐LDL‐Induced Endothelial Progenitor Cell Senescence via Gria2 and cAMP Pathways

**DOI:** 10.1111/jcmm.70682

**Published:** 2025-07-10

**Authors:** Jinjian Wu, Guotao Lu, Zhou Luo, Meng Cai, Qiankun He, Jie Su, Jianfeng Liu, Rong Wang, Chunyan Kuang

**Affiliations:** ^1^ Department of Cardiology Guizhou Provincial People's Hospital Guiyang China; ^2^ Department of Cardiology, Integrated Traditional Chinese and Western Medicine Hospital Liangshan Yi Autonomous Prefecture Xichang Sichuan China; ^3^ College of Veterinary Medicine Northwest A&F University Yangling China; ^4^ Department of Cardiology Ziyang Hospital of Traditional Chinese Medicine Ziyang Sichuan China; ^5^ Department of Personnel, Science and Education Baoshan Second People's Hospital Baoshan Yunnan China; ^6^ Department of Infection Management, People's Hospital of Xichang City Liangshan Yi Autonomous Prefecture Xichang Sichuan China; ^7^ Second Clinical Medical College Guizhou University of Traditional Chinese Medicine Guiyang Guizhou China

**Keywords:** cAMP signalling pathway, endothelial progenitor cells, Gria2, in‐stent restenosis, senescence, Xinmailong

## Abstract

In‐stent restenosis (ISR) following percutaneous coronary intervention (PCI) is a critical clinical issue, often arising from endothelial injury and impaired repair mechanisms. Endothelial progenitor cells (EPCs), derived from bone marrow, play a key role in vascular health, but their function diminishes with aging and exposure to oxidised low‐density lipoprotein (ox‐LDL). This study investigates the potential of Xin‐Mai‐Long (XML), a traditional Chinese medicine used for chronic congestive heart failure, to delay EPC senescence and dysfunction induced by ox‐LDL. Our findings demonstrate that XML administration significantly attenuates ox‐LDL‐induced EPC senescence and dysfunction. RNA sequencing identified Gria2 as a crucial gene downregulated by ox‐LDL and restored by XML. Overexpression of Gria2 in EPCs similarly protected against ox‐LDL‐induced damage. Further analysis using Gene Ontology (GO) and KEGG enrichment revealed that the cAMP signalling pathway is significantly activated in response to XML and Gria2 overexpression. Notably, inhibition of cAMP with cis‐Epoxysuccinic Acid (CESA) diminished the protective effects of XML and Gria2, underscoring the importance of this pathway. In vivo experiments using a rat carotid balloon injury model showed that both XML administration and transplantation of Gria2‐overexpressing EPCs reduced vascular damage. These results suggest that XML mitigates EPC senescence and dysfunction by upregulating Gria2 and activating the cAMP signalling pathway, offering a promising therapeutic strategy for managing ISR after PCI.

## 1 Introduction

Cardiovascular diseases (CVDs) are the leading cause of mortality in the world [1]. CVDs kills more people than any other disease, accounting for nearly one‐third of deaths worldwide [2]. This disease encompasses a wide range of pathologies, including deep vein thrombosis and atherosclerosis, which is the main cause of CVD [2]. Despite tremendous advances in the diagnosis and treatment of CVD over the past 20 years, CVD mortality is still high [3, 4, 5]. It is estimated that the number of people currently suffering from CVD in China is around 330 million [6], making it the most common cause of death in the country. Vascular aging is an independent risk factor for age‐related diseases [7]. The process of vascular aging is characterised by a progressive deterioration in vascular structure and function, and the main pathological changes involves medial intima thickening, increased fibrosis and decreased endothelial function [8, 9, 10].

Xinmailong (XML), approved by China's National Medical Products Administration (NMPA) for treating cardiac insufficiency, addresses a condition often tied to vascular pathologies such as atherosclerosis and endothelial dysfunction. These impairments are key contributors to in‐stent restenosis (ISR) following percutaneous coronary intervention (PCI), a persistent clinical challenge driven by delayed or impaired endothelial repair [11]. Endothelial progenitor cells (EPCs) are critical for vascular regeneration, but their senescence—accelerated by factors like oxidised low‐density lipoprotein (ox‐LDL)—compromises this process, exacerbating ISR risk. Given XML's documented cardiovascular benefits, including improved cardiac function and potential anti‐inflammatory effects, we hypothesize that it may reduce EPC senescence and enhance endothelial repair [12]. This suggests a novel therapeutic role for XML in managing ISR and related vascular injuries, warranting further investigation.

Endothelial progenitor cells (EPCs), firstly isolated from peripheral blood in 1997 [13], were identified as a subset of bone marrow‐derived progenitor cells. EPCs can migrate into the peripheral blood circulation and quickly proliferate and differentiate into mature ECs that express the surface markers CD31, CD34, CD133 and VEGFR‐2 [14, 15]. EPC‐based cell therapy is considered a promising approach for rapid re‐endothelialisation of vascular injury sites after coronary artery stenting, potentially preventing ISR and late stent thrombosis [16]. In response to vascular injury, EPCs can be mobilised to the injury site, resulting in endothelial repair and contributing to increased reendothelialisation associated with decreased neointima formation [17, 18]. Therefore, EPCs are promising resources for stem cell‐based therapies [19]. In addition, EPCs are also used as predictive biomarkers to assess cardiovascular disease risk [20]. However, with ageing‐associated senescence, the numbers and function of EPCs decline, which leads to impaired capacities to contribute to vascular repair and regeneration [21, 22]. Specifically, cellular ageing led to a decrease in cell yield, dysfunction and engraftment of cells after transplantation of EPCs [23, 24]. Therefore, controlling risk factors and delaying EPC senescence are key strategies for enhancing their function and quantity to achieve rapid re‐endothelialisation after coronary artery stenting.

XML is a bioactive composite extracted from Periplaneta (a species of cockroach) and approved by the China State Food and Drug Administration (CFDA) in 2006 for the treatment of cardiac dysfunction. A systematic study of XML's chemical composition, and quantitative analysis was performed, and results showed that 29 compounds were identified using high‐performance liquid chromatography (HPLC) and gas chromatograph–mass spectrometer (GC–MS) from the active fraction of XML [25, 26]. Future investigation indicated that XML contains four main active ingredients: adenosine, inosine, protocatechuic acid and pyroglutamate dipeptide [27]. Oxidised low‐density lipoprotein (ox‐LDL) is taken up by EPCs in a receptor‐dependent manner. The level of ox‐LDL increases in patients with coronary artery disease or diabetes and serves as an independent predictor for future cardiac events in these patients [28, 29]. Several studies have shown that ox‐LDL functions as an important factor that adversely affects the growth and bioactivity of EPCs by triggering apoptosis, inducing cell ossification, and inhibiting endothelialization [30, 31, 32]. It has also been demonstrated that ox‐LDL also accelerated the senescence of EPCs, leading to cellular dysfunction [33, 34]. In this study, we investigated whether XML could attenuate ox‐LDL‐induced EPCs senescence in vitro and balloon‐induced vascular endothelial injury in vivo.

## 2 Materials and Methods

### 2.1 Isolation and Cultivation of Endothelial Progenitor Cells

Endothelial progenitor cells (EPCs) were isolated from the bone marrow of Sprague–Dawley rats. Total bone marrow was extracted from the femurs and tibias of rats using DMEM/F12 medium (Thermo Fisher, Cat# 11320033) in a sterile environment. Mononuclear cells were isolated via density gradient centrifugation using Ficoll‐Paque (GE Healthcare, Cat# 17‐1440‐02), then seeded into culture dishes and cultured in DMEM/F12 supplemented with 10% fetal bovine serum (FBS, Thermo Fisher, Cat# 26140079), 50 μg/mL endothelial cell growth supplement (EGS, Sigma–Aldrich, Cat# E2759), 100 U/mL penicillin (Sigma–Aldrich, Cat# P3032) and 100 μg/mL streptomycin (Sigma–Aldrich, Cat# S9137). This medium, referred to as complete DMEM/F12, was used for general EPC cultivation and subsequent experiments (e.g., SA‐β‐gal staining, EdU assay) unless otherwise specified. On Day 4, non‐adherent cells were thoroughly washed away, and the adherent cells were subsequently cultured in fresh growth medium. After 4 days of culture, spindle‐shaped EPCs appeared and were further expanded for subsequent experiments. All experiments were conducted using EPCs at passages 2 to 4 to ensure the maintenance of primary cell characteristics and minimise senescence due to prolonged culture.

### 2.2 Gria2‐Overexpression in EPCs

Gria2 was amplified by PCR from cDNA and cloned into pLVX‐Puro (GenScript, Cat# Z03528). The construct was then packaged into lentivirus using HEK293T cells transfected with Lipofectamine 3000 (Thermo Fisher, Cat# L3000015). EPCs were transduced and selected with 2 μg/mL puromycin (Sigma–Aldrich, Cat# P8833) for 7 days. Control cells were transduced with pLVX‐Puro empty vector (Takara Bio, Cat. No. 632164) or pLVX‐Puro‐GFP lentivirus.

### 2.3 Rat Carotid Balloon Injury Model

Male Sprague–Dawley rats (8–10 weeks old, 250–300 g) were purchased from Charles River Laboratories, Wilmington, MA, USA, and housed at 22°C ± 2°C with a 12‐h light/dark cycle, 50%–60% humidity, and ad libitum access to food and water. A high‐fat diet (40% fat, Research Diets Inc., Cat# D12492) was provided for 4 weeks prior to surgery to induce atherogenesis. Rats were anaesthetised with 2% isoflurane (inhalation, Covetrus, Cat# 029404) and placed supine. A midline cervical incision exposed the left common carotid artery, where a 2F Fogarty balloon catheter (Edwards Lifesciences, Cat# NL2EMB40) was inserted via arteriotomy, inflated to 1.5 atm and withdrawn three times to denude the endothelium. The artery was ligated distally, and the incision closed with 5–0 sutures. Sham‐operated rats underwent vessel exposure without balloon injury. Post‐surgery, GFP‐overexpressing or Gria2‐overexpressing EPCs (1 × 10^6^ cells/rat, tail vein) were administered daily for 3 days. Rats were euthanized 14 days post‐injury via CO_2_ inhalation.

### 2.4 Flow Cytometry

Peripheral blood samples from Sprague–Dawley rats were incubated with mouse anti‐rat VEGFR‐2 antibody (Abcam, Cat# ab2349, 1:200) and rabbit anti‐rat CD34 antibody (Abcam, Cat# ab81289, 1:200) at room temperature for 30 min. Isotype controls (mouse IgG, Abcam, Cat# ab37355, 1:200; rabbit IgG, Abcam, Cat# ab37415, 1:200) were run in parallel to set fluorescence thresholds and exclude non‐specific binding. Samples were then treated with goat anti‐mouse IgG H&L (Alexa Fluor 594, Abcam, Cat# ab150116, 1:500) and donkey anti‐rabbit IgG H&L (Alexa Fluor 488, Abcam, Cat# ab150073, 1:500) for 30 min at room temperature. Red blood cells were lysed with NH4Cl solution (Sigma–Aldrich, Cat# A9434) for 10 min, followed by washing with PBS. Cells were fixed in 1% formaldehyde (Sigma–Aldrich, Cat# F8775) and analysed on a Navios Flow Cytometer (Beckman Coulter) using Kaluza Analysis Software v2.1. Gating thresholds were established based on isotype control fluorescence (< 2% positive), and the percentage of CD34+ and VEGFR‐2+ cells was quantified from 10,000 events per sample.

### 2.5 Cell Staining

After 4 days in culture, bone marrow‐derived EPCs underwent extensive washing and were incubated with 10 μg/mL Dil‐ac‐LDL (Invitrogen, Cat# L3484) for 4 h at 37°C. Post‐incubation, cells were fixed with 4% phosphate‐buffered paraformaldehyde (PFA, Thermo Fisher, Cat# 28906) for 10 min. Subsequently, they were stained with FITC‐conjugated UEA‐1‐lectin (Sigma‐Aldrich, Cat# L9006) at 37°C for 4 h. Cells positive for both DiI‐Ac‐LDL and lectin were identified as EPCs and enumerated.

### 2.6 XML Administration and Treatment Groups

EPCs were exposed to oxidised low‐density lipoprotein (ox‐LDL, Sigma‐Aldrich, Cat# L34357) at a concentration of 15 mg/mL and treated with various doses of Xin‐Mai‐Long (XML) from a commercial supplier: high‐dose (1 mg/mL), middle‐dose (0.5 mg/mL) and low‐dose (0.25 mg/mL). Cells were incubated for 24 h to assess the effects of XML on EPC senescence and dysfunction.

### 2.7 Senescence‐Associated β‐Galactosidase (SA‐β‐Gal) Staining

To assess senescence‐associated β‐galactosidase (SA‐β‐gal) activity as a biomarker for cellular senescence, we utilised the Senescence Detection Kit (Abcam, Cat# ab65351) following the manufacturer's protocol. Quiescent EPCs cultured to 70% confluence in 6‐well plates were exposed to various experimental conditions. The cells were rinsed with phosphate‐buffered saline (PBS, Thermo Fisher, Cat# 10010049), fixed in 2% paraformaldehyde (PFA, Thermo Fisher, Cat# 28906) for 3 min at room temperature, washed again and incubated at 37°C for 16 h with fresh SA‐β‐gal stain solution. Subsequently, the cells were photographed, and the percentage of SA‐β‐gal‐positive perinuclear blue cells was quantified by counting five randomly selected fields.

### 2.8 Scratching Wound Healing Assay

EPCs were initially seeded into 6‐well plates and allowed to grow until reaching confluence. Subsequently, a standardised scratch was generated in each well using a cell scratcher (Sigma‐Aldrich, Cat# S2123), and the plates were washed with EGM‐2 medium (Lonza, Cat# CC‐3162) to remove detached cells and debris. The scratched cell monolayers were then maintained in EGM‐2 medium, which contains basal medium and growth factors per the manufacturer's formulation (e.g., VEGF, bFGF) without additional FBS or cytokines, and incubated at 37°C in a humidified atmosphere. EGM‐2 was specifically used in this assay to optimise endothelial cell migration, differing from the complete DMEM/F12 used in other experiments. Throughout the incubation period, images of the migrating cells were periodically captured using a light microscope.

### 2.9 Quantitative Real‐Time PCR

Total RNA was extracted from EPCs using TRIzol reagent (Invitrogen, Cat# 15596026) according to the manufacturer's protocol. First‐strand cDNA was synthesised in a 20 μL reaction containing 1 μg of total RNA, 1 mM dNTPs (Thermo Fisher, Cat# R0191), 200 U of SuperScript III Reverse Transcriptase (Thermo Fisher, Cat# EP0742), and 100 pmol of random hexamer oligonucleotides (Thermo Fisher, Cat# SO142), using a T100 Thermal Cycler (Bio‐Rad). cDNA concentration was measured with a NanoDrop 2000 spectrophotometer (Thermo Fisher), typically yielding 20–50 ng/μL. For quantitative real‐time PCR (RT‐qPCR), 1 μL of cDNA (approximately 20–50 ng) was amplified in a 20 μL reaction with Power SYBR Green Master Mix (Thermo Fisher, Cat# A25742) on a CFX96 Real‐Time System (Bio‐Rad). Cycling conditions were as follows: 95°C for 10 min, followed by 40 cycles of 95°C for 15 s and 60°C for 1 min. Relative gene expression was calculated using the 2^−ΔΔ*Ct*^ method, normalised to GAPDH. Primers were designed to target rat ( *Rattus norvegicus* ) genes, as EPCs were derived from Sprague–Dawley rats.

The following primers were used to detect the expression of specific genes:

Gria2: FP 5′‐TTCTCCTGTTTTATGGGGACTGA‐3′ RP: 5′‐CCCTACCCGAAATGCACTGTA‐3′,

PKA: FP 5′‐AGATCGTCCTGACCTTTGAG‐3′ RP 5′‐TCCACACAGCCTTGTTCGTAGC‐3′,

Creb5: FP 5′‐GCAAGGTCCAAACCTCAGCAAC‐3′ RP 5′‐TGTCCGATGGTGCTCATGTTCC‐3′,

Adcy10: FP 5′‐CCTCACAAGTGGTGTCAGACTG‐3′ RP 5′‐CAACTCAGTGGTGAAGGTCAGG‐3′,

Erg: FP 5′‐GGACAGACTTCCAAGATGAGCC‐3′ RP 5′‐CCACACTGCATTCATCAGGAGAG‐3′,

Ibsp: FP 5′‐AATGGAGACGGCGATAGTTCCG‐3′ RP 5′‐GGAAAGTGTGGAGTTCTCTGCC‐3′,

Olr59: FP 5′‐AGGGGCTATGCTTAAACTGAAAGA‐3′ RP 5′‐TTCTACAGTCAGCCGCAAGG‐3′,

Nefl: FP 5′‐CGAAGACGCCACTAACGAGA‐3′ RP 5′‐CAACTCAGTGGTGAAGGTCAGG‐3′.

### 2.10 Western Blot Analysis

EPCs were initially washed with cold PBS (Thermo Fisher, Cat# 10010049) and subsequently lysed in a harvesting buffer composed of 20 mM Tris–HCl (pH 7.5, Sigma–Aldrich, Cat# T1503), 20 mM p‐nitrophenyl phosphate (Sigma–Aldrich, Cat# P4744), 1 mM EGTA (Sigma–Aldrich, Cat# E3889), 50 mM sodium fluoride (Sigma–Aldrich, Cat# S7920), 50 μM sodium orthovanadate (Sigma–Aldrich, Cat# S6508) and 5 mM benzamidine (Sigma–Aldrich, Cat# B6506). Following lysis, the cells were scraped, homogenised on ice, and centrifuged at 4000 × *g* at 4°C for 5 min. The resulting supernatants were collected and subjected to a secondary high‐speed centrifugation at 12,000 rpm. These supernatants were subsequently stored at −80°C until further analysis. Aliquots of each lysate were separated by 10% SDS‐PAGE (Thermo Fisher, Cat# NP0323BOX) and transferred onto nitrocellulose membranes (Bio‐Rad, Cat# 1620112). The membranes were then probed with primary antibodies (Table S1). After washing, membranes were incubated with HRP‐conjugated secondary antibodies (Thermo Fisher, Cat# 31460), and signals were detected using an ECL detection system (Thermo Fisher, Cat# 32109).

### 2.11 RNA Sequencing and Bioinformatics Analysis

Total RNA was extracted from EPCs using TRIzol reagent (Invitrogen, Cat# 15596026) following the manufacturer's protocol. RNA integrity was assessed using an Agilent 2100 Bioanalyzer (Agilent Technologies), with RNA Integrity Number (RIN) values > 7.0 deemed acceptable. Sequencing libraries were constructed using the NEBNext Ultra II RNA Library Prep Kit for Illumina (New England Biolabs, Cat# E7770) according to the manufacturer's instructions, involving poly(A) mRNA enrichment, fragmentation and adapter ligation. Libraries were sequenced on an Illumina NovaSeq 6000 platform (Illumina) with 150 bp paired‐end reads, generating approximately 20 million reads per sample. Raw reads were quality‐checked using FastQC v0.11.9 and trimmed with Trimmomatic v0.39 to remove adapters and low‐quality bases (Phred score < 20). Clean reads were aligned to the rat reference genome ( *Rattus norvegicus* , Ensembl Rnor_6.0) using STAR v2.7.9a with default parameters. Gene expression was quantified with featureCounts v2.0.1, and differentially expressed genes (DEGs) were identified using DESeq2 v1.34.0 in R, defined as genes with a log2 fold change > 1 and adjusted *p*‐value < 0.05 (Benjamini–Hochberg correction). Gene Ontology (GO) and Kyoto Encyclopedia of Genes and Genomes (KEGG) pathway enrichment analyses were performed using DAVID v6.8, with the rat genome as the background. Significant terms were selected based on a *p*‐value < 0.05.

### 2.12 EdU Cell Proliferation Assay

EPCs were cultured on coverslips within a 6‐well plate and treated with 10 μM EdU (5‐ethynyl‐2′‐deoxyuridine, Abcam, Cat# ab146186) for 5 h to assess proliferation. EdU incorporation was detected using the Click‐iT EdU Alexa Fluor 488 Imaging Kit (Thermo Fisher, Cat# C10337) following the manufacturer's protocol. Briefly, incorporated EdU was labelled via a copper‐catalysed click reaction with Alexa Fluor 488 azide, producing green fluorescence. After staining, coverslips were mounted with ProLong Gold Antifade Mountant (Thermo Fisher, Cat# P36930), and images were acquired using a fluorescence microscope (e.g., Zeiss Axio Observer) with a 488 nm excitation filter to visualise and quantify EdU‐positive cells.

### 2.13 Measurement of Serum Lipid Levels

Blood samples were collected from the rats via tail vein puncture into serum separator tubes and centrifuged at 3000 × *g* for 10 min at 4°C to obtain serum. Serum levels of total cholesterol (TC; Roche, Cat# 03039773), triglycerides (TG; Roche, Cat# 03039774), high‐density lipoprotein (HDL; Roche, Cat# 03039775) and low‐density lipoprotein (LDL; Roche, Cat# 03039776) were measured using an automated biochemical analyser (Roche, Cat# COBAS INTEGRA 400 Plus) according to the manufacturer's protocols.

### 2.14 Enzyme‐Linked Immunosorbent Assay (ELISA)

Serum levels of senescence‐associated secretory phenotype (SASP) cytokines and reactive oxygen species (ROS) were measured using specific ELISA kits according to the manufacturers' protocols. Rat TNF‐α was quantified with R&D Systems Cat# RTA00, IL‐1 with R&D Systems Cat# RLB00, HMGB1 with R&D Systems Cat# DY1690, MMP‐3 with R&D Systems Cat# MMP300, and ROS with OxiSelect ROS Assay Kit (Cell Biolabs, Cat# STA‐347). Assays were performed in duplicate, and absorbance was read at 450 nm (corrected at 540 nm) using a microplate reader (Bio‐Rad, Model 680). Concentrations were calculated from standard curves to assess inflammation and oxidative stress.

### 2.15 Histological Analysis

Carotid arteries were harvested, fixed in 10% neutral‐buffered formalin (Thermo Fisher, Cat# 5737) for 24 h, and embedded in paraffin. Five serial sections (5 μm thick) per sample were cut at 100 μm intervals from the injury site and stained with haematoxylin and eosin (H&E, Sigma–Aldrich, Cat# GHS132) to evaluate intimal hyperplasia. Stained sections were imaged using a light microscope (e.g., Olympus BX51) at 20× magnification. Intimal hyperplasia was defined as an intima‐to‐media (I/M) ratio > 0.5, where the intima and media thicknesses were measured using ImageJ software (NIH, v1.53). For each section, three random fields were analysed, and the average I/M ratio was calculated per sample for quantitative assessment.

### 2.16 Statistical Analysis

Data are presented as mean ± SEM. Normality was assessed via Shapiro–Wilk test, and variance homogeneity via Levene's test. For normal data, Student's *t*‐test (two groups) or one‐way ANOVA with Tukey's post hoc test (multiple groups) was used. Non‐normal data were analysed with Kruskal–Wallis test; unequal variance used Welch's ANOVA. *p*‐value correction employed Bonferroni method. Analyses were performed using GraphPad Prism 8.0.

## 3 Results

### 3.1 Cultivation and Identification of EPCs

**FIGURE 1:**
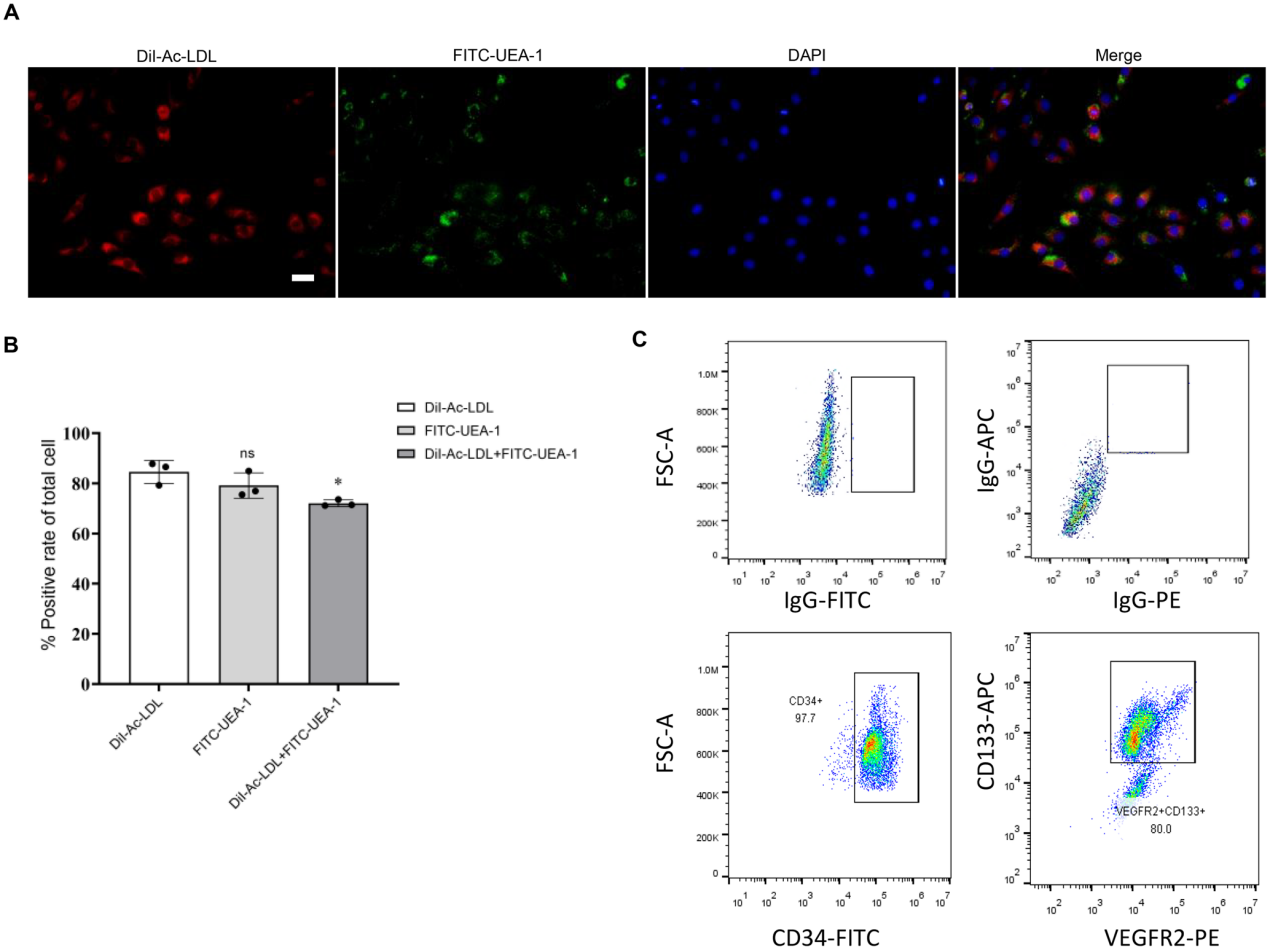
Cultivation and identification of EPCs. (A, B) Representative fluorescence images showing EPCs double positive for DiI‐Ac‐LDL incorporation and lectin binding, confirming their identity. Scale bar = 50 μm. Quantified the proportion of the double positive for Dil‐Ac‐LDL and FITC‐UEA‐1 in (B) , *p < 0.05. (C) Flow cytometry results of peripheral blood mononuclear cell‐derived endothelial progenitor cells' FACS analysis showed that EPCs highly expressed CD34 and VEGFR2.

EPCs were isolated from the bone marrow of Sprague–Dawley rats as previously described [15]. Briefly, Total bone marrow mononuclear cells (BM‐MNCs) were isolated and cultured for 4 days that resulted in a spindle‐shaped morphology, and looked like ‘cobblestone’. EPCs can incorporate Dil‐Ac‐LDL and bind lectin, and BM‐MNCs double positive for DiI‐Ac‐LDL and lectin binding were labelled as ex vivo‐expanded EPCs [18]. After staining, images were obtained by fluorescence microscopy. Our data show that most cells were double‐positive (Figure 1A,B). In addition, the surface markers CD34 and VEGFR2 were tested by flow cytometry to confirm the purity of EPCs (Figure 1C).

### 3.2 XML Attenuates Ox‐LDL Induced EPCs Senescence and Dysfunction

**FIGURE 2:**
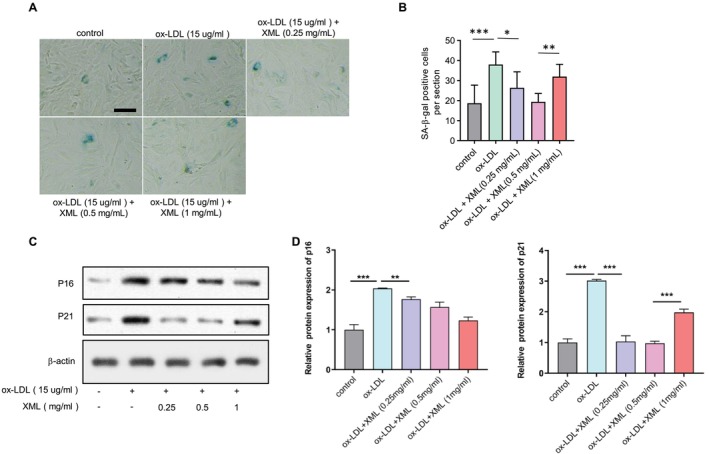
XML attenuates ox‐LDL induced EPC senescence. (A) SA‐β‐gal staining of EPCs treated with ox‐LDL and XML doses. Scale bar = 50 μm. (B) Quantification of SA‐β‐gal‐positive cells from 5 random fields per sample (*n* = 3, mean ± SEM). *p < 0.05, **p < 0.01, ***p < 0.001. (C) Western blot analysis of pro‐senescence proteins p16 and p21 in EPCs treated with ox‐LDL and XML. (D) Quantification of Western blot results, indicating that XML treatment decreases the levels of p16 and p21 proteins (*n* = 3). **p < 0.01, ***p < 0.001. Error bars represent mean ± SEM.

EPCs were exposed to oxidised low‐density lipoprotein (ox‐LDL, 15 mg/mL) and incubated with or without high‐dose (1 mg/mL), middle‐dose (0.5 mg/mL) or low‐dose (0.25 mg/mL) XML. We assessed β‐galactosidase (SA‐β‐gal) activity to determine the senescence of EPCs. We found that ox‐LDL successfully increased SA‐β‐gal positive cell numbers and middle‐dose XML administration restored the aging phenotype of EPCs (Figure 2A,B). To further confirm the anti‐senescence effects of XML, we used western blot to assess the protein level of pro‐senescence proteins p16 and p21. Following treatment of EPCs with ox‐LDL, both p16 and p21 protein levels increased and XML treatment decreased them (Figure 2C,D). These data suggest that XML attenuates cellular senescence induced by ox‐LDL in EPCs.

**FIGURE 3:**
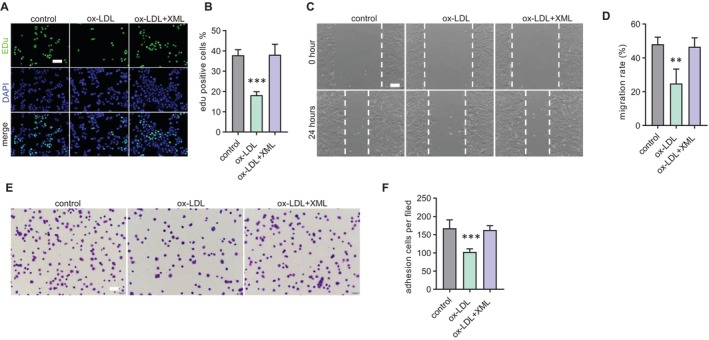
XML attenuates ox‐LDL‐induced EPCs dysfunction. (A) Representative images of EdU staining for cell proliferation in EPCs treated with ox‐LDL and XML, showing increased proliferation in XML‐treated groups. Scale bar = 50 μm. (B) Quantification of EdU‐positive cells, demonstrating enhanced proliferation with XML treatment (*n* = 3). ***p < 0.001. Error bars represent mean ± SEM. (C) Representative images of scratch wound healing assay images of EPC migration in ox‐LDL and XML‐treated groups. Scale bar = 100 μm. (D) Quantification of wound closure, indicating improved migration ability with XML treatment (*n* = 3). **p < 0.01. Error bars represent mean ± SEM. (E) Representative images of cell adhesion assay images showing increased adhesion of EPCs with XML treatment. Scale bar = 50 μm. (F) Quantification of adherent cells, confirming enhanced adhesion in XML‐treated EPCs (*n* = 3). ***p < 0.001. Error bars represent mean ± SEM.

A reduction in cell proliferation, cell adhesion and decrease in cell migration are representative aging phenotypes of cells [23]. To investigate the effects of XML on senescent EPCs function, we firstly examined the proliferation of EPCs cultured with XML via Edu staining. Compared with ox‐LDL group, XML administration remarkably increased the proportion of Edu‐positive cells (Figure 3A,B). A scratch wound healing assay was performed to assess the effect of XML on EPCs migration ability. The results demonstrated a significant promotion of senescent EPCs wound healing compared with ox‐LDL group (Figure 3C,D). Subsequently, EPCs matrix adhesion assay was used to measure cell adhesion. EPCs exhibited a significant increase in the number of adhesive cells following XML administration angst ox‐LDL (Figure 3E,F). EPCs function analysis showed that XML can improve function of senescent EPCs.

### 3.3 XML Activates Gria2 in Senescent EPCs Induced by Ox‐LDL

To explore the underlying mechanisms of protective effects of XML angst ox‐LDL‐induced damage, we performed RNA‐sequencing (RNA‐seq) analysis on three EPCs groups (EPCs, EPCs + ox‐LDL; and EPCs + ox‐LDL + XML). RNA‐seq data showed than 256 genes were downregulated after ox‐LDL treatment, and 237 genes were upregulated following XML administration (Figure S1A). Ox‐LDL associated downregulated and XML associated upregulated expression genes were intersected and 40 candidate gene were identified (Figure S1B). We next validated the top five genes (Erg, Ibsp, Olr59, Nefl and Gria2) by RT‐qPCR. The results of RT‐qPCR showed that the expression trend of these genes were consistent with RNA‐seq data (Figure S1C). Among the top five genes, the change level of Gria2 gene was the most obvious upon XML administration. We also used western blot to assessed the protein level of Gria2. Gria2 protein was suppressed by ox‐LDL and restored following XML administration (Figure S1D,E).

### 3.4 XML and Gria2‐Overexpression Attenuates Ox‐LDL‐Induced EPCs Senescence and Dysfunction Through Activating cAMP Signalling Pathway

**FIGURE 4:**
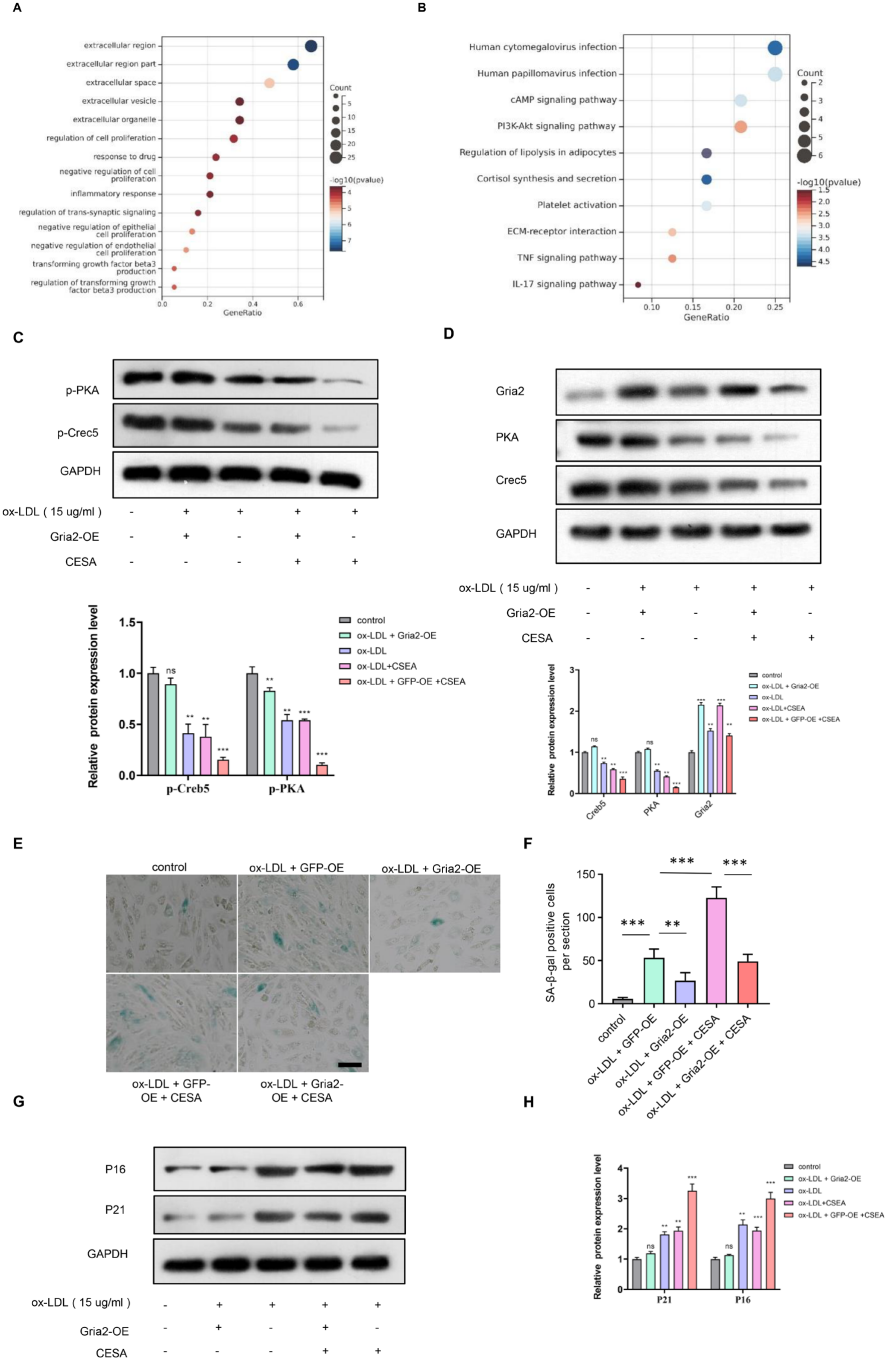
XML and Gria2‐overexpression activate cAMP signalling pathway. (A) GO analysis of DEGs (log2 fold change > 1, *p* < 0.05) from EPCs + ox‐LDL vs. EPCs (downregulated) and EPCs + ox‐LDL + XML vs. EPCs + ox‐LDL (upregulated). (B) KEGG analysis of DEGs as above, highlighting cAMP pathway. (C) Western blot analysis of phosphorylated PKA and CREB in EPCs treated with XML, Gria2‐overexpression and CESA. (D) Quantification of Western blot results, showing enhanced cAMP signalling with XML and Gria2‐overexpression (*n* = 3). **p < 0.01, ***p < 0.001. Error bars represent mean ± SEM. (E) Representative images of SA‐β‐gal staining showing that inhibition of cAMP by CESA compromises the anti‐senescence effects of XML and Gria2‐overexpression. Scale bar = 50 μm. (F) Quantification of SA‐β‐gal positive cells, confirming the involvement of cAMP signalling in EPC protection (*n* = 3). **p < 0.01, ***p < 0.001. Error bars represent mean ± SEM. (G) Western blot analysis of pro‐senescence proteins p16 and p21 in EPCs treated with ox‐LDL, Gria2‐overexpression and cAMP inhibitor. (H) Quantification of Western blot results (*n* = 3). *p < 0.05, **p < 0.01, ***p < 0.001. Error bars represent mean ± SEM.

**FIGURE 5:**
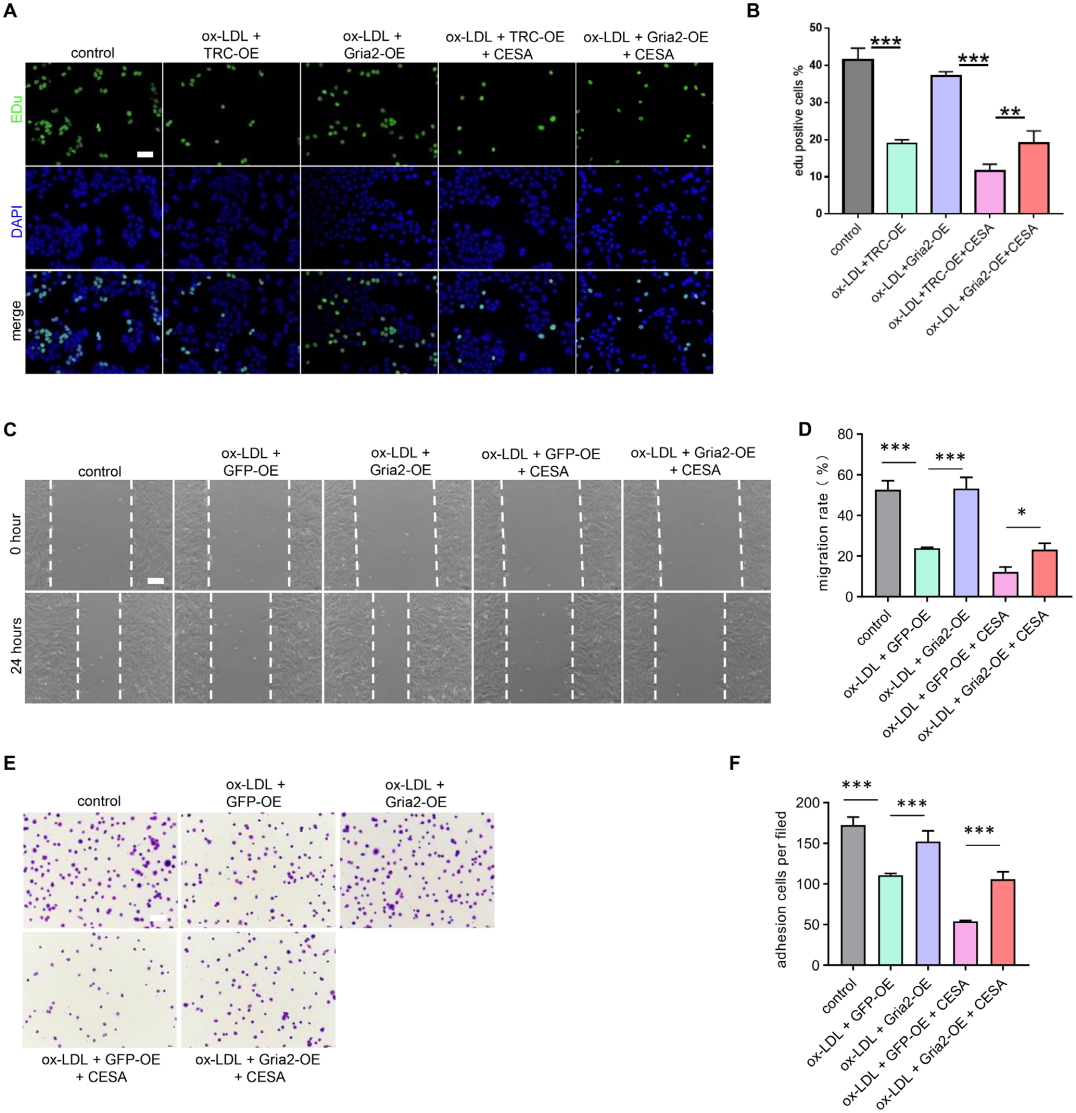
Gria2‐overexpression attenuates ox‐LDL‐induced EPCs dysfunction through activating cAMP signalling pathway. (A) Representative images of EdU staining for cell proliferation in EPCs treated with ox‐LDL, Gria2‐overexpression and cAMP inhibitor. Scale bar = 50 μm. (B) Quantification of EdU‐positive cells, demonstrating enhanced proliferation with Gria2‐overexpression treatment, which was blocked by cAMP inhibition (*n* = 3). **p < 0.01, ***p < 0.001. Error bars represent mean ± SEM. (C) Representative images of scratch wound healing assay images of EPC migration in ox‐LDL, Gria2‐overexpression, and cAMP inhibitor groups. Scale bar = 100 μm. (D) Quantification of wound closure, indicating improved migration ability with Gria2‐overexpression treatment, which was blocked by cAMP inhibition (*n* = 3). *p < 0.05, ***p < 0.001. Error bars represent mean ± SEM. (E) Representative images of cell adhesion assay images of EPCs treated with ox‐LDL, Gria2‐overexpression and cAMP inhibitor. Scale bar = 50 μm. (F) Quantification of adherent cells, confirming that enhanced adhesion by Gria2‐overexpression was blocked by cAMP inhibition (*n* = 3). ***p < 0.001. Error bars represent mean ± SEM.

Gene Ontology analysis (GO) revealed enrichment in cell senescence, cell proliferation and inflammatory response (Figure 4A). Kyoto Encyclopedia of Genes and Genomes (KEGG) enrichment analysis suggested that the differentially expressed genes were mainly involved in the cAMP signalling pathway (Figure 4B). The activation of the cAMP signalling pathway was the most significant, and previous studies have demonstrated that the cAMP signalling pathway is involved in cell senescence. So, we next investigated the involvement of the cAMP signalling pathway in the protection of XML and Gria2. To further investigate the role of Gria2 in XML‐protected EPCs senescence and dysfunction, we constructed a stable Gria2‐overexpressing EPCs with a lentivirus system, and control cells were transfected with lentivirus containing GFP (Figure S1F). Cis‐Epoxysuccinic acid (CESA), a succinate receptor agonist inhibiting cAMP levels, was used to block the cAMP signalling pathway. Phosphorylated PKA and CREB5 were detected to measure the activity of the cAMP signalling pathway by western blot. Results showed that XML administration and Gria2‐overexpression enhanced the phosphorylated level of PKA and CREB5, and CESA abolished it (Figure 4C,D). SA‐β‐gal assay showed that inhibition of cAMP by CESA compromised the anti‐senescence effects of XML administration and Gria2‐overexpression (Figure 4E,F). Pro‐senescence proteins p16 and p21 were also increased in CESA‐treated EPCs compared with XML administration and Gria2‐overexpression groups (Figure 4G,H). In addition, cAMP inhibition via CESA also blocked cell function improvement acquired from XML administration and Gria2‐overexpression as evaluated by Edu staining (Figure 5A,B), scratch wound healing assay (Figure 5C,D) and matrix adhesion assay (Figure 5E,F). These results indicated that XML and Gria2‐overexpression attenuate ox‐LDL‐induced EPCs senescence and dysfunction through activating the cAMP signalling pathway.

### 3.5 XML Administration and Transplantation of Gria2‐Overexpression‐EPCs Reduce Vascular Damage In Vivo

**FIGURE 6:**
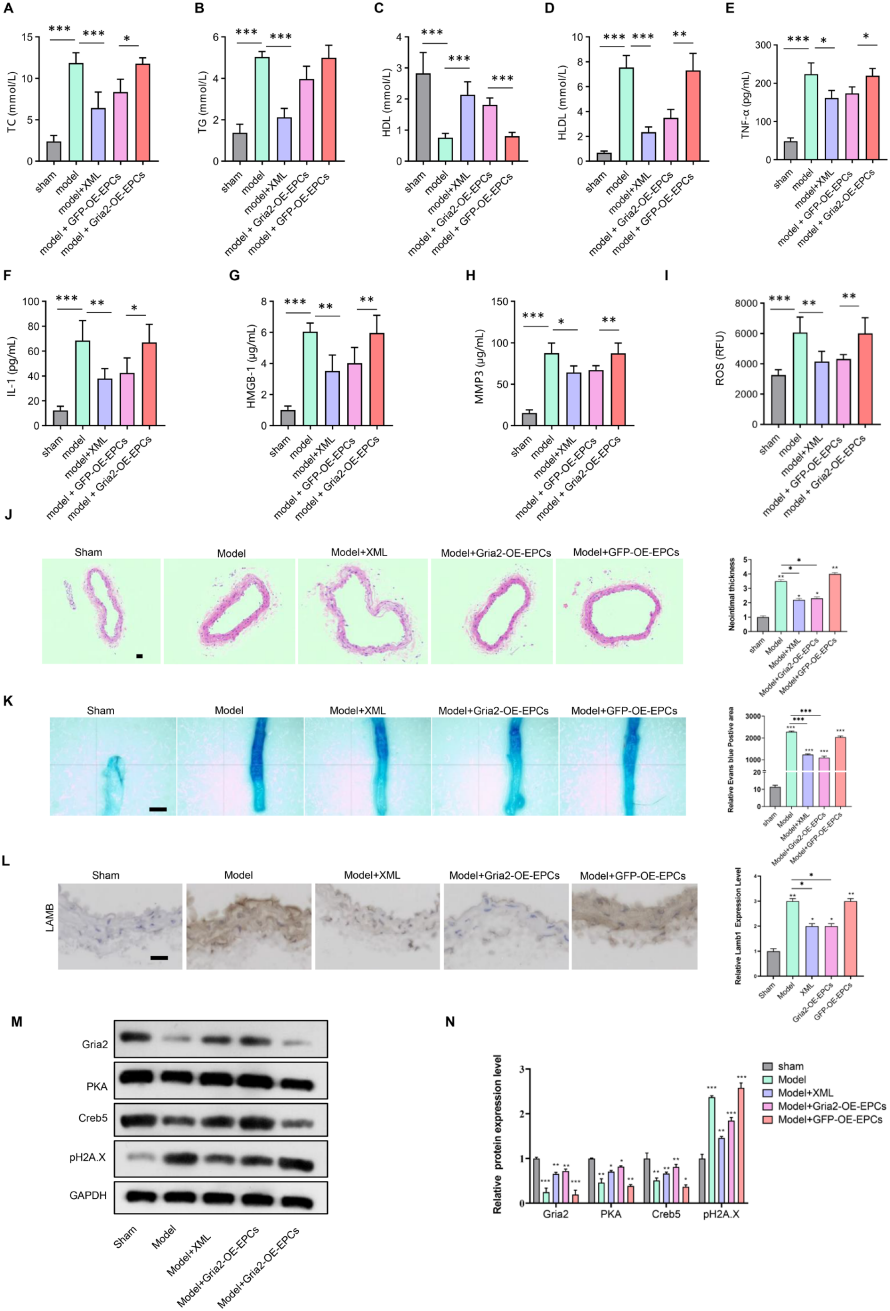
XML administration and Gria2‐overexpression‐EPCs reduce vascular damage in vivo. (A) Serum lipid levels of total cholesterol (TC) in model rats treated with XML and Gria2‐overexpression‐EPCs (*n* = 5). *p < 0.05, ***p < 0.001. Error bars represent mean ± SEM. (B) Serum triglycerides (TG) levels in the same groups (*n* = 5). ***p < 0.001. Error bars represent mean ± SEM. (C) High‐density lipoprotein (HDL) levels, showing restoration with XML and Gria2‐overexpression‐EPCs (*n* = 5). ***p < 0.001. Error bars represent mean ± SEM. (D) Low‐density lipoprotein (LDL) levels, indicating decreased levels in treated groups (*n* = 5). **p < 0.01, ***p < 0.001. Error bars represent mean ± SEM. (E–H) ELISA results of senescence‐associated secretory phenotype (SASP) cytokines (TNF‐α, IL‐1, HMGB1 and MMP‐3) in model rats (*n* = 5). *p < 0.05, **p < 0.01, ***p < 0.001. Error bars represent mean ± SEM. (I) Measurement of serum reactive oxygen species (ROS) levels, showing reduction with XML and Gria2‐overexpression‐EPCs (*n* = 5). **p < 0.01, ***p < 0.001. Error bars represent mean ± SEM. (J) Haematoxylin and eosin (H&E) staining of carotid arteries, indicating reduced intimal hyperplasia in treated groups. Scale bar = 100 μm. The relative neointimal thickness was quantified and is shown in the right panel (*n* = 5). **p < 0.01. Error bars represent mean ± SEM. (K) Evans blue staining of carotid arteries, illustrating reduced vascular endothelial injury with XML and Gria2‐overexpression‐EPCs treatment. Scale bar = 100 μm. The relative area of Evans blue‐positive regions was quantified and is presented in the right panel (*n* = 5). **p < 0.01, ***p < 0.001. Error bars represent mean ± SEM. (L) Immunohistochemistry staining of LMNB1, indicating reduced senescence of carotid arteries in treated groups. Scale bar = 100 μm. The relative expression level of LMNB1 was quantified and is shown in the right panel (*n* = 5). **p < 0.01. Error bars represent mean ± SEM. (M) Western blot analysis of cAMP activation in EPCs treated with ox‐LDL, Gria2‐overexpression and cAMP inhibitor. (N) Quantification of Western blot results (*n* = 3). *p < 0.05, **p < 0.01, ***p < 0.001. Error bars represent mean ± SEM.

To investigate the protective effects of XML and Gria2‐overexpression in vivo, we used a rat carotid balloon injury model (see Materials and Methods, ‘Rat Carotid Balloon Injury Model’). XML (1 mg/kg/day, i.p.) and Gria2‐overexpressing EPCs (1 × 10^6^ cells/rat, tail vein) were administered daily for 3 days post‐injury. After 14 days, the rats were euthanized and their serum were collected to measure serum lipids. The results showed that serum levels of TC, TG and HDL were elevated, and serum levels of HLDL were decreased in model rat group. XML administration and transplantation of Gria2‐overexpression‐EPCs decreased serum levels of TC, TG and HDL, and restored HLDL levels (Figure 6A–D). Furthermore, our ELISA assay results showed that the serum levels of senescence‐associated secretory phenotype (SASP) cytokines (TNF‐α, IL‐1, HMGB1 and MMP‐3) and ROS were increased in model rat group and XML administration and transplantation of Gria2‐overexpression‐EPCs also restored them (Figure 6E–I). We used hyperplasia by haematoxylin and eosin (HE) staining to evaluate the intimal hyperplasia, and our results demonstrated severe intimal hyperplasia in model groups, which was hindered by XML administration and transplantation of Gria2‐overexpression‐EPCs (Figure 6J). Evans blue staining showed that rats in the model groups exhibited serious vascular endothelial injury, and XML administration and transplantation of Gria2‐overexpression‐EPCs alleviated vascular endothelial injury (Figure 6K). Moreover, Masson staining revealed pronounced collagen fibre deposition in the vascular walls of model groups, which was markedly reduced following XML treatment and Gria2‐overexpression‐EPC transplantation (Figure S2A,B). The expression of LaminB1 (LMNB1) decreased during the aging process. We detected the decreased expression of LMNB1 in the model group by immunohistochemistry, and the expression recovered after XML administration and transplantation of Gria2‐overexpression‐EPCs (Figure 6L). Pro‐senescence proteins p16 and p21 were markedly increased in the model group, and this upregulation was reversed by XML administration and transplantation of Gria2‐overexpression‐EPCs (Figure S2C,D). We also confirmed that cAMP signalling pathway was activated through detecting the expression of PKA and its downstream CREB5 by western blot (Figure 6M,N). Moreover, XML administration and Gria2‐overexpression enhanced the phosphorylated levels of PKA and CREB5, while these effects were abolished by CESA treatment (Figure S2E,F). These data suggested that XML and Gria2 contribute to the reduction in balloon injury in vivo.

## 4 Discussion

In this study, we showed that XML significantly delayed ox‐LDL‐induced senescence of EPCs and restored the proliferation, migration and adhesion of senescent EPCs. Mechanistic investigations revealed that XML upregulates the expression of Glutamate Ionotropic Receptor AMPA Type Subunit 2 (Gria2) in EPCs, thereby activating the downstream cyclic adenosine monophosphate (cAMP) signalling pathway to mediate its anti‐senescence effects. Cis‐Epoxysuccinic acid, an inhibitor of cAMP signalling, reduced the expression of Gria2 in the senescent EPCs and blocked the protective function of XML and Gria2‐OE‐EPCs. In an atherosclerotic rat carotid artery injury model, administration of XML or transplantation of Gria2‐overexpressing EPCs ameliorated dyslipidemia and reduced oxidative stress, markers of cellular senescence and cellular DNA damage. It promoted vascular re‐endothelialisation while inhibiting vascular intimal hyperplasia. These findings suggest that the XML‐mediated Gria2/cAMP axis represents a potential target for preventing and treating EPC dysfunctions associated with cardiovascular disease.

Reendothelialization is one of the determinants of the development of neointima formation [35], and essential in preventing restenosis after percutaneous coronary intervention (PCI) and transplantation of autologous EPCs contribute to vascular repair and neovascularization in different limb ischemia and myocardial infarction animal models [36]. Recent years the unique value of traditional chinese medicine has been widely recognised and concerned by the international community. XML, a bioactive composite extracted from Periplaneta, has been widely used in the treatment of cardiovascular diseases such as congestive heart failure [37, 38]. Additional studies have shown that XML compounds have protective effects against cardiovascular injury though anti‐inflammatory and vasodilatory activities [39]. Investigation on heart indicated that XML could reduce myocardial injury by attenuating ROS production, apoptosis and inflammatory responses [40]. Another study on heart showed that XML effectively attenuates adriamycin‐induced cardiomyopathy via inhibits cellular autophagy by activating the PI3K/Akt signalling pathway [39]. These studies suggest that XML has important anti‐inflammatory, anti‐oxidative stress and autophagy‐regulating functions, which are beneficial for the treatment of atherosclerosis, increasing circulating EPCs and contribute to reendothelialization and inhibiting endothelial proliferation.

Dyslipidemia plays a critical role in endothelial dysfunction and atherosclerosis progression [41]. The balloon injury model combined with a high‐fat diet led to elevated TC, TG and LDL‐C levels while decreasing HDL, mimicking human atherosclerosis. This effect is primarily attributed to endothelial injury‐induced lipid accumulation, inflammatory activation and impaired lipid metabolism. Endothelial disruption increases permeability to lipoproteins, allowing LDL‐C infiltration and oxidation, which drives foam cell formation and sustained vascular inflammation [42]. Additionally, lipoprotein lipase (LPL) activity at injury sites may contribute to localised TG hydrolysis, enhancing hepatic VLDL synthesis and perpetuating dyslipidemia. Inflammation further exacerbates this imbalance by downregulating hepatic LDL receptors, reducing LDL‐C clearance [43]. Moreover, HDL dysfunction occurs due to inflammation‐mediated alterations in apoA‐I synthesis and reverse cholesterol transport capacity, which further aggravates lipid deposition [44].

GRIA2 encodes an AMPA‐selective ionotropic glutamate receptor subunit (also known as GLUR2), affects cell membrane calcium permeability and plays an important role in the excitatory synaptic conduction of fast synaptic currents at AMPA receptors in the mammalian brain [45]. A recent study showed that overexpression of GRIA2 promoted the proliferation and migration of VSMCs [46]. cAMP, a secondary messenger transmitting a variety of hormonal and neurotransmitter signals in different cells, is required for cellular homeostasis and plays crucial roles in many processes. Increased levels of cAMP can reverse some of the adverse effects of aging in senescence‐related diseases [47]. In our study, XML administration and transplantation of Gria2‐OE‐EPCs were shown to be effective in delaying senescence of EPCs and reducing atherosclerotic balloon injury via cAMP signalling. These results together support the important role of XML in anti‐endothelial cell senescence and anti‐vascular injury. Despite this study revealing a protective effect of XML against ox‐LDL‐induced senescent EPCs, there are limitations in using a rat carotid balloon injury model which fails to fully mimic the clinical situation of coronary artery restenosis in humans. So, it is necessary to validate it through additional clinic trial. In addition, how XML administration and Gria2‐OE activate cAMP signalling is not illustrated precisely and more molecular biology experiments are needed to address it.

Furthermore, our study expands the current understanding of Gria2 function beyond its established role in neuronal excitatory synaptic transmission and reveals its involvement in regulating EPC biology and vascular remodelling processes. Interestingly, the Gria2/cAMP axis may also influence other interconnected anti‐aging pathways. cAMP has been shown to regulate the activity of SIRT1, a key regulator of cellular senescence and lifespan [48]. Moreover, PKA may also inhibit mTORC1 activity by phosphorylating the upstream regulatory factors of mTORC1, Raptor and TSC2, improving various age‐related diseases such as cardiovascular diseases, neurodegenerative diseases and metabolic diseases [49]. Additionally, the cAMP/PKA pathway can activate FOXO transcription factors, which play crucial roles in stress resistance, cell cycle regulation and longevity [50]. Therefore, the Gria2/cAMP axis may exert pleiotropic effects by coordinating multiple aging‐related pathways, ultimately contributing to the observed improvements in dyslipidemia, reduction of senescence‐associated secretory phenotype (SASP) factors and oxidative stress, downregulation of cellular senescence markers and alleviation of DNA damage in the carotid artery tissues of rats treated with XML or injected with Gria2‐overexpressing EPCs. Importantly, these interventions also promoted re‐endothelialisation and inhibited intimal abnormal hyperplasia of the injured carotid arteries, highlighting the potential therapeutic significance of targeting EPC senescence in vascular endothelial repair and ISR prevention.

While this study demonstrates the protective effects of XML as a composite, it did not evaluate the individual contributions of its four main active ingredients: adenosine, inosine, protocatechuic acid and pyroglutamate dipeptide. Identifying the most effective component(s) in attenuating EPC senescence and dysfunction could refine therapeutic applications. Due to time and resource constraints, such experiments were beyond the scope of this study; however, future investigations will explore these ingredients separately to elucidate their specific roles.

In conclusion, this is a novel study demonstrating that XML attenuates ox‐LDL‐induced endothelial progenitor cell senescence and dysfunction via activation of the cAMP signalling pathway. Overexpression of Gria2 could also mimic the protective function of XML. XML administration and transplantation of Gria2‐OE‐EPCs reduce vascular damage in vivo in a rat carotid balloon injury model.

## Author Contributions

**Jinjian Wu:** conceptualization (equal), data curation (equal), formal analysis (equal), investigation (equal), writing – original draft (equal). **Guotao Lu:** investigation (equal). **Zhou Luo:** investigation (equal), methodology (equal). **Meng Cai:** investigation (equal), methodology (equal). **Qiankun He:** investigation (equal), methodology (equal). **Jie Su:** investigation (equal), methodology (equal). **Jianfeng Liu:** data curation (equal), methodology (equal). **Rong Wang:** data curation (equal), project administration (equal). **Chunyan Kuang:** funding acquisition (equal), validation (equal), writing – original draft (equal).

## Ethics Statement

This animal study was reviewed and approved by the Ethics Committee of Guizhou Provincial People's Hospital (Approval No. 20230191).

## Conflicts of Interest

The authors declare no conflicts of interest.

## Supporting information


**Figure S1.** XML Activates Gria2 in Senescent EPCs Induced by ox‐LDL. (A) RNA‐seq analysis of EPCs treated with ox‐LDL and XML, identifying differentially expressed genes. (B) Venn diagram showing overlap of downregulated genes by ox‐LDL and upregulated genes by XML. (C) RT‐qPCR validation of top 5 candidate genes, confirming the RNA‐seq results. (*n* = 3). ***p < 0.001. Error bars representmean ± SEM. (D) Western blot analysis of Gria2 protein levels in EPCs treated with ox‐LDL and XML. (E, F) Quantification of Western blot results, showing that XML restores Gria2 protein levels. (*n* = 3). ***p < 0.001. Error bars represent mean ± SEM. (G) Confirmation of successful lentiviral overexpression of Gria2 in EPCs via qPCR. ***p < 0.001. Error bars represent mean ± SEM.
**Figure S2.** XML and Gria2‐overexpression Attenuate Vascular Injury and Senescence in vivo. (A) Masson staining of carotid arteries, showing pronounced collagen fibre deposition in model groups, which was alleviated by XML and Gria2‐overexpression‐EPCs treatment. Scale bar = 100 μm. (B) Quantification of collagen deposition area from Masson staining images (*n* = 5). ***p < 0.001. Error bars represent mean ± SEM. (C) Western blot analysis of p16 and p21 expression in carotid tissues, indicating reduced senescence markers following XML and Gria2‐overexpression‐EPCs treatment. (D) Quantification of p16 and p21 expression levels (*n* = 3). ***p < 0.001. Error bars represent mean ± SEM. (E) Western blot analysis of phosphorylated PKA and CREB5 in carotid arteries from different treatment groups. (F) Quantification of p‐PKA and p‐CREB5 expression, showing activation of the cAMP pathway by XML and Gria2‐overexpression‐EPCs, and its suppression by CESA treatment (*n* = 3). *p < 0.05, **p < 0.01, ***p < 0.001. Error bars represent mean ± SEM.

**Table S1.** List of primary antibodies used for Western blotting and immunohistochemistry.

## Data Availability

The authors have nothing to report.
