# Supplementary material for: XML Attenuates Ox‐LDL‐Induced Endothelial Progenitor Cell Senescence via Gria2 and cAMP Pathways
Source: J Cell Mol Med. 2025 Jul 10;29(13):e70682. doi: 10.1111/jcmm.70682 (PMC12244391; doi:10.1111/jcmm.70682)
Supplement: Supplementary file 1 — Figure S1. XML Activates Gria2 in Senescent EPCs Induced by ox‐LDL. (A) RNA‐seq analysis of EPCs treated with ox‐LDL and XML, identifying differentially expressed genes. (B) Venn diagram showing overlap of downregulated genes by ox‐LDL and upregulated genes by XML. (C) RT‐qPCR validation of top 5 candidate genes, confirming the RNA‐seq results. (n = 3). ***p < 0.001. Error bars representmean ± SEM. (D) Western blot analysis of Gria2 protein levels in EPCs treated with ox‐LDL and XML. (E, F) Quantification of Western blot results, showing that XML restores Gria2 protein levels. (n = 3). ***p < 0.001. Error bars represent mean ± SEM. (G) Confirmation of successful lentiviral overexpression of Gria2 in EPCs via qPCR. ***p < 0.001. Error bars represent mean ± SEM. Figure S2. XML and Gria2‐overexpression Attenuate Vascular Injury and Senescence in vivo. (A) Masson staining of carotid arteries, showing pronounced collagen fibre deposition in model groups, which was alleviated by XML and Gria2‐overexpression‐EPCs treatment. Scale bar = 100 μm. (B) Quantification of collagen deposition area from Masson staining images (n = 5). ***p < 0.001. Error bars represent mean ± SEM. (C) Western blot analysis of p16 and p21 expression in carotid tissues, indicating reduced senescence markers following XML and Gria2‐overexpression‐EPCs treatment. (D) Quantification of p16 and p21 expression levels (n = 3). ***p < 0.001. Error bars represent mean ± SEM. (E) Western blot analysis of phosphorylated PKA and CREB5 in carotid arteries from different treatment groups. (F) Quantification of p‐PKA and p‐CREB5 expression, showing activation of the cAMP pathway by XML and Gria2‐overexpression‐EPCs, and its suppression by CESA treatment (n = 3). *p < 0.05, **p < 0.01, ***p < 0.001. Error bars represent mean ± SEM. [file JCMM-29-e70682-s002.zip › Supplementary Figurelegends.docx]

**Supplementary figure 1.** XML Activates Gria2 in Senescent EPCs Induced by ox-LDL.

(A) RNA-seq analysis of EPCs treated with ox-LDL and XML, identifying differentially expressed genes.

(B) Venn diagram showing overlap of downregulated genes by ox-LDL and upregulated genes by XML.

(C) RT-qPCR validation of top 5 candidate genes, confirming the RNA-seq results. (n = 3). ***p < 0.001. Error bars representmean ± SEM.

(D) Western blot analysis of Gria2 protein levels in EPCs treated with ox-LDL and XML.

(E,F) Quantification of Western blot results, showing that XML restores Gria2 protein levels. (n = 3). ***p < 0.001. Error bars represent mean ± SEM.

(G) Confirmation of successful lentiviral overexpression of Gria2 in EPCs via qPCR. ***p < 0.001. Error bars represent mean ± SEM.

**Supplementary Figure 2. XML and Gria2-Overexpression Attenuate Vascular Injury and Senescence In *vivo*.**

(A) Masson staining of carotid arteries, showing pronounced collagen fiber deposition in model groups, which was alleviated by XML and Gria2-overexpression-EPCs treatment. Scale bar = 100 µm.

(B) Quantification of collagen deposition area from Masson staining images (n = 5). ***p < 0.001. Error bars represent mean ± SEM.

(C) Western blot analysis of p16 and p21 expression in carotid tissues, indicating reduced senescence markers following XML and Gria2-overexpression-EPCs treatment.

(D) Quantification of p16 and p21 expression levels (n = 3). ***p < 0.001. Error bars represent mean ± SEM.

(E) Western blot analysis of phosphorylated PKA and CREB5 in carotid arteries from different treatment groups.

(F) Quantification of p-PKA and p-CREB5 expression, showing activation of the cAMP pathway by XML and Gria2-overexpression-EPCs, and its suppression by CESA treatment (n = 3). *p < 0.05, **p < 0.01, ***p < 0.001. Error bars represent mean ± SEM.
