# Supplementary material for: XML Attenuates Ox‐LDL‐Induced Endothelial Progenitor Cell Senescence via Gria2 and cAMP Pathways
Source: J Cell Mol Med. 2025 Jul 10;29(13):e70682. doi: 10.1111/jcmm.70682 (PMC12244391; doi:10.1111/jcmm.70682)
Supplement: Supplementary file 2 — Table S1. List of primary antibodies used for Western blotting and immunohistochemistry. [file JCMM-29-e70682-s001.docx]

Table S1. List of primary antibodies used for Western blotting and immunohistochemistry.

| **Primary Antibody** | **Company** | **Product Number** | **Dilution Concentration** |
| --- | --- | --- | --- |
| p16 | proteintech | 10883-1-AP | 1:1000 |
| p21 | proteintech | 10355-1-AP | 1:2000 |
| Gria2 | proteintech | 11994-1-AP | 1:2000 |
| Phosphorylated PKA | CST | 9621 | 1:1000 |
| CREB5 | proteintech | 14196-1-AP | 1:500 |
| LaminB1 (LMNB1) | proteintech | 12987-1-AP |  |
| GAPDH | proteintech | 60004-1-Ig | 1:5000 |
| β-actin | proteintech | 66009-1-Ig | 1:20000 |
| Phosphorylated Creb5 | CST | 80864 | 1:1000 |
| pH2A.X | CST | 2577 | 1:1000 |
